# Phased Haplotype Resolution of the *SLC6A4* Promoter Using Long-Read Single Molecule Real-Time (SMRT) Sequencing

**DOI:** 10.3390/genes11111333

**Published:** 2020-11-12

**Authors:** Mariana R. Botton, Yao Yang, Erick R. Scott, Robert J. Desnick, Stuart A. Scott

**Affiliations:** 1 Department of Genetics and Genomic Sciences, Icahn School of Medicine at Mount Sinai, New York, NY 10029, USA; yaoy@stanford.edu (Y.Y.); erick.scott@mssm.edu (E.R.S.); robert.desnick@mssm.edu (R.J.D.); 2 Cells, Tissues and Genes Laboratory, Hospital de Clínicas de Porto Alegre, Porto Alegre 90035-903, RS, Brazil; 3 Department of Pathology, Stanford University, Palo Alto, CA 94305, USA; 4 Stanford Medicine Clinical Genomics Program, Stanford Health Care, Stanford, CA 94305, USA; 5 Sema4, Stamford, CT 06902, USA

**Keywords:** *SLC6A4*, selective serotonin reuptake inhibitors (SSRI), long-read sequencing, single molecule real-time (SMRT) sequencing, Pacific Biosciences (PacBio), pharmacogenetics, pharmacogenomics, haplotype phasing

## Abstract

The *SLC6A4* gene has been implicated in psychiatric disorder susceptibility and antidepressant response variability. The *SLC6A4* promoter is defined by a variable number of homologous 20–24 bp repeats (5-HTTLPR), and long (L) and short (S) alleles are associated with higher and lower expression, respectively. However, this insertion/deletion variant is most informative when considered as a haplotype with the rs25531 and rs25532 variants. Therefore, we developed a long-read single molecule real-time (SMRT) sequencing method to interrogate the *SLC6A4* promoter region. A total of 120 samples were subjected to *SLC6A4* long-read SMRT sequencing, primarily selected based on available short-read sequencing data. Short-read genome sequencing from the 1000 Genomes (1KG) Project (~5X) and the Genetic Testing Reference Material Coordination Program (~45X), as well as high-depth short-read capture-based sequencing (~330X), could not identify the 5-HTTLPR short (S) allele, nor could short-read sequencing phase any identified variants. In contrast, long-read SMRT sequencing unambiguously identified the 5-HTTLPR short (S) allele (frequency of 0.467) and phased *SLC6A4* promoter haplotypes. Additionally, discordant rs25531 genotypes were reviewed and determined to be short-read errors. Taken together, long-read SMRT sequencing is an innovative and robust method for phased resolution of the *SLC6A4* promoter, which could enable more accurate pharmacogenetic testing for both research and clinical applications.

## 1. Introduction

Antidepressants are the third most commonly prescribed drug class in the United States, surpassed only by antihyperlipidemics and analgesics [1]. In addition to depression, selective serotonin reuptake inhibitors (SSRIs) are used to treat obsessive-compulsive disorder (OCD), panic disorder, posttraumatic stress disorder and anxiety disorders; however, efficacy among the different SSRIs has been estimated at only ~65% across treated patients [2]. In an effort to improve antidepressant efficacy, pharmacogenetic studies have recently been undertaken to guide treatment based on common variants in genes involved in SSRI pharmacokinetics (e.g., *CYP2D6* and *CYP2C19*) and pharmacodynamics (*SLC6A4*) [3].

The *SLC6A4* gene at chromosome 17q11.2 encodes the serotonin (5-HT) transporter (5-HTT), which mediates 5-HT reuptake and is the major target of SSRI antidepressants. Expression of 5-HTT is directly correlated with 5-HT transporter function, which has been implicated as a susceptibility gene for several psychiatric disorders (e.g., affective disorders, schizophrenia, anxiety, autism, depression, suicide, OCD, and addiction [4]) as well as a pharmacogenetic determinant of interindividual SSRI response variability [5,6,7,8]. The polymorphic *SLC6A4* promoter is composed of a variable number (11–24) of tandem repeat units (5-HTTLPR), which are 20–24 bp in length and highly homologous. The common 5-HTTLPR alleles implicated in SSRI response are the long (L; 16 repeats) and short (S; 14 repeats) haplotypes, which are defined by a 43 bp insertion/deletion polymorphism (rs4795541) and are strongly associated with higher and lower gene expression, respectively [9,10,11,12]. In addition to these two alleles, extra-long (XL; 17–24 repeats) and extra-short (XS; 11–13 repeats) promoter alleles, have also been identified with low frequencies in the general population [13,14,15,16,17,18]; however, their effects on gene expression are still being elucidated [19].

In addition to the 5-HTTLPR L>S variant (rs4795541), two *SLC6A4* promoter sequence variants (rs25531 (c.-1936A>G), rs25532 (c.-1760C>T)) have also been reported to influence 5-HTT expression; however, they are most informative when considered as a haplotype with 5-HTTLPR [20,21]. The rs25531 (A>G) variant is located in the sixth repeat unit and can occur on either the L or S 5-HTTLPR haplotype. Notably, when this variant is found on the L haplotype (“L_G_”), it is associated with lower gene expression, similar to the S allele [20,21]. The rs25532 (C>T) variant is located ~150 bp from rs25531 in the µ element. The presence of the T allele at this position also is associated with lower gene expression on both the L and S haplotypes [21]. Given the potential clinical significance of *SLC6A4* and the importance of phasing multiple variants across the polymorphic repetitive promoter region, we developed a novel long-read single molecule real-time (SMRT) sequencing method to comprehensively characterize *SLC6A4* promoter haplotypes.

## 2. Materials and Methods

### 2.1. Samples and Publicly Available Data

A total of 120 independent Coriell samples were subjected to long-read SMRT sequencing at the polymorphic *SLC6A4* promoter region, primarily selected based on the availability of orthogonal short-read sequencing data. Among these samples, low-depth short-read genome sequencing (WGS) data (~5X) was available for 32 samples from the 1000 Genomes (1KG) Project; high-depth short-read WGS data (~45X) were available for 68 samples from the Centers for Disease Control and Genetic Testing Reference Material Coordination Program (GeT-RM) pharmacogenetic testing program [22]; and high-depth short-read capture-based panel data (~330X) were available for 21 samples from an internal dataset. DNA samples were acquired from the Coriell Biorepository (Camden, NJ, USA), which are summarized in Supplemental Table S1.

1KG variant call format files (Phase 3 release v5a) were downloaded from the National Center for Biotechnology Information (NCBI) FTP server [23]. GeT-RM Illumina FASTQ and BAM files from were downloaded from the GeT-RM website and the European Nucleotide Archive (http://www.ebi.ac.uk/ena/data/view/PRJEB19931). 1KG Phase 3 and GeT-RM reference calls were evaluated using GATK Haplotype caller (version 4.1.3, -ERC GVCF -L 17:28563658-28564683), genotypes were called using PyPGx (v0.1.12, bam2vcf gatk 17:28563658-28564683 hg19) [24]. GeT-RM and high-depth short-read capture-based genotypes were also called using an internal Genome Analysis Tool Kit (GATK) best practices pipeline. GNU Parallel was used to increase the rate of data processing [25].

### 2.2. PCR Amplification of the SLC6A4 Promoter

Four overlapping primer sets (with a common forward primer) were used to amplify the *SLC6A4* promoter region (Table 1), which included universal oligonucleotide tags for subsequent barcoding and SMRT sequencing. Long-range PCR reactions were performed in 20 µL containing ~100 ng of DNA, 1X SequalPrep^TM^ Reaction buffer (Invitrogen, Carlsbad, CA, USA), 0.5 µM of forward and reverse primers, and 1.8 units of SequalPrep^TM^ polymerase. Amplification conditions were identical for all primer sets and consisted of an initial denaturation step at 94 °C for 2 min followed by 10 amplification cycles (94 °C for 10 s, 63.6 °C for 30 s, and 68 °C for 90 s), another 20 amplification cycles (94 °C for 10 s, 63.6 °C for 30 s, and 68 °C for 90 s + 20 s/cycle), and a final extension at 72 °C for 5 min. These products were used as templates for barcoding prior to SMRT sequencing.

### 2.3. SLC6A4 Promoter Sample Barcoding and Pooling

First round amplification products were used as template for a subsequent PCR that incorporated forward and reverse barcodes using forward (5′-[barcode]-ATGGGTTCCAGAGTCAATC-3′) and reverse (5′-[barcode]-GAAAGGTCTGGAGTCTTGAT–3′) primers (Supplemental Table S2). The PCR conditions for this reaction were identical to the first round PCR described above. Agencourt^®^ AMPure^®^ XP beads were used to purify all barcoded PCR amplicons, which were quantified by Nanodrop 1000. Following purification and quantitation, sample library pools were generated with equal molecule quantities of PCR amplicons using the following formula to calculate the required volume of each amplicon [26]:

$$V\left(i\right)=\frac{M}{n\times C\left(i\right)}$$

where *M* is the total mass of pooled PCR amplicons, *n* is the total number of samples, *V*(*i*) is the volume of each PCR amplicon, and *C*(*i*) is the concentration of each amplicon. A total of 4000 ng of pooled PCR amplicons was submitted for SMRT sequencing.

### 2.4. Single Molecule Real-Time (SMRT) Sequencing

SMRT sequencing was executed according to the P6-C4 Pacific Biosciences protocol with a movie collection time of 180 min on the Sequel instrument, as per the manufacturer’s instructions and as previously described [26]. SMRT sequencing analysis included demultiplexing, alignment, quality score recalibration, and variant calling. Raw sequencing data in FASTQ format were demultiplexed using NGSutils, sequencing reads were aligned using BWA-MEM, and variant calling was performed using GATK [26].

### 2.5. Sanger Sequencing Confirmation

Selected samples were validated by orthogonal Sanger sequencing using the following primer set: FWD 5′-CTTTGCGTTTTCTGTTGCCCT-3′; REV 5′-CCCAGCAGGAGCCTATTGTT-3′. These primers generated a 1026 bp amplicon using the same PCR conditions described above. PCR fragments were analyzed using the Applied Biosystems 3730xl DNA Analyzer, and chromatogram traces were evaluated using NG_011747.2 as a reference sequence and Chromas v2.6.6 (South Brisbane, Australia).

## 3. Results

### 3.1. SLC6A4 Promoter Short-Read Sequencing

Genotype results from the interrogated *SLC6A4* promoter region identified by short-read WGS (1KG and GeT-RM) and capture-based enrichment sequencing are summarized in Supplemental Table S2. The 5-HTTLPR (rs4795541), rs25531 and rs25532 variant sites were either not detectable or incorrectly genotyped among the 1KG WGS cohort, the GeT-RM WGS cohort, and the short-read capture-based sequencing cohort in 32/32 (100%), 60/68 (88%) and 17/21 (81%) samples and 87/96 (91%), 85/204 (42%) and 34/63 (54%) variant sites, respectively (Supplemental Table S2).

### 3.2. SLC6A4 Promoter Long-Read SMRT Sequencing

**Figure 1:**
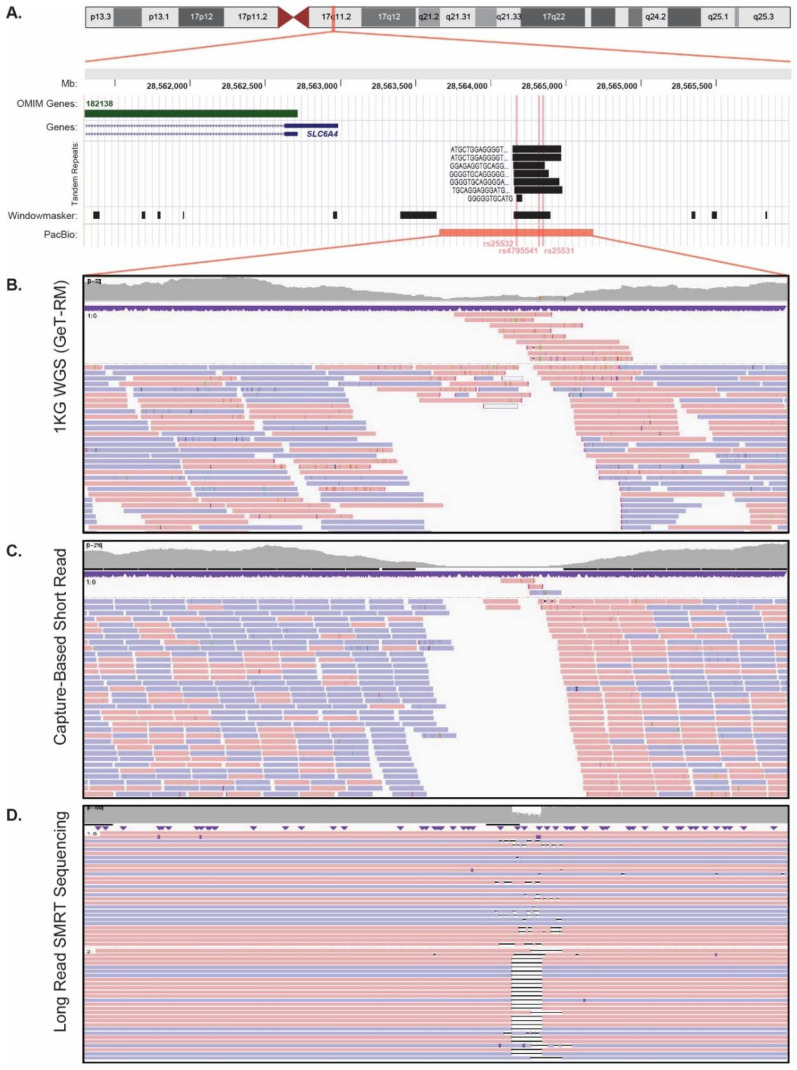
Representative *SLC6A4* promoter sequencing of the NA12156 Coriell sample using short-read (Genetic Testing Reference Material Coordination Program (GeT-RM) genome sequencing (WGS), and capture-based sequencing) and long-read single molecule real-time (SMRT) sequencing. Note the inability to interrogate the repetitive *SLC6A4* promoter region (**A**) by two short-read sequencing approaches (WGS (**B**) and capture-based sequencing (**C**)), in contrast to the 5-HTTLPR short (S) allele detection and haplotype phasing accomplished by long-read SMRT sequencing (**D**).

**Figure 2:**
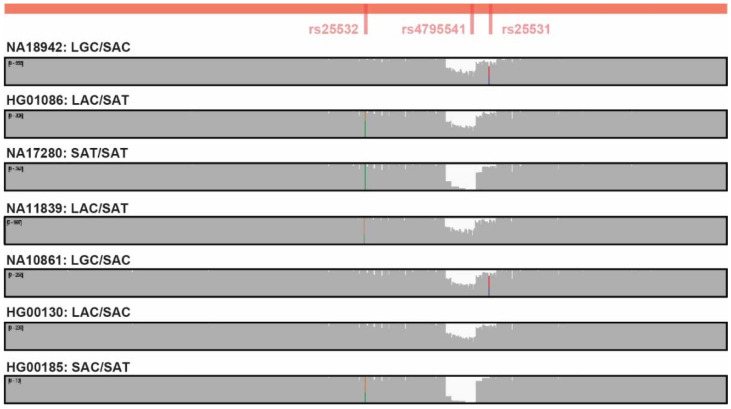
Representative *SLC6A4* promoter sequencing results from seven Coriell DNA samples using long-read SMRT sequencing: NA18942 (LGC/SAC), HG01086 (LAC/SAT), NA17280 SAT/SAT, NA11839 (LAC/SAT), NA10861 (LGC/SAC), HG00130 (LAC/SAC), HG00185 (SAC/SAT).

All 120 samples were subjected to long-read SMRT sequencing across the polymorphic *SLC6A4* promoter, and a representative sample with both short- and long-read sequencing data available is illustrated in Figure 1. Long-read SMRT sequencing results of additional *SLC6A4* diplotypes are illustrated in Figure 2. In contrast to the short-read sequencing datasets, the 5-HTTLPR short (S) allele (rs4795541) was detected by long-read SMRT sequencing in 79 samples, resulting in a MAF of 0.467 (112/240). In addition, the *SLC6A4* rs25531 and rs25532 variants were detectable in all samples and their MAFs were 0.113 (27/240) and 0.088 (21/240), respectively. In addition, long-read SMRT sequencing detected two samples with extra-long (XL) 5-HTTLPR alleles that were not detected by short-read sequencing. Six additional *SLC6A4* promoter sequence variants were also detected and phased by long-read SMRT sequencing (Supplemental Table S3), which were inconsistently detected among the short-read sequencing datasets.

In addition to being more accurate at this locus than short-read sequencing, long-read SMRT sequencing also unambiguously phased the polymorphic *SLC6A4* promoter in all samples, including complex compound heterozygous diplotypes (Figure 2). The three clinically significant *SLC6A4* promoter variants (5-HTTLPR (rs4795541), rs25531, rs25532) were phased and the identified diplotypes and haplotype frequencies are summarized in Supplemental Table S2 and Table 2, respectively.

### 3.3. SLC6A4 Promoter Long-Read SMRT Sequencing Sanger Validation

Accuracy of long-read SMRT sequencing at the polymorphic *SLC6A4* promoter was further evaluated by orthogonal Sanger sequencing. Six DNA samples were selected based on their representative diplotypes: LAC/LGC (HG00276, NA12336), LGC/SAC (HG01190), LAC/SAT (NA07000), LAC/XLAC (NA18855), and SAC/XLAC (NA19174). Sanger sequencing confirmed all variant alleles identified by long-read SMRT sequencing, including the short (S) and extra-long (XL) 5-HTTLPR alleles that were not detected by short-read sequencing (Supplemental Table S4 and Figure S1).

### 3.4. SLC6A4 Promoter Long-Read SMRT Sequencing Precision

Precision of long-read SMRT sequencing was measured by subjecting reference material samples with identified *SLC6A4* variant alleles (*n* = 15) to intra-run (repeatability) and inter-run (reproducibility) triplicate testing (i.e., 3:1:1 validation). In summary, the intra- and inter-run genotype and diplotype concordances for the 15 control samples were both 100% (225/225 genotypes; 150/150 haplotypes) (Supplemental Table S5).

## 4. Discussion

Given the emerging clinical significance of *SLC6A4* in interindividual SSRI response variability and the repetitive architecture of the homologous *SLC6A4* promoter, we developed a novel long-read SMRT sequencing assay using the PacBio platform to comprehensively characterize *SLC6A4* promoter region haplotypes. Our innovative method enabled the phased resolution of complex *SLC6A4* promoter diplotypes, which was not possible using short-read WGS data (~5X and ~45X) or high-depth capture-based short-read sequencing data (~330X). This novel pharmacogenetic application of long-read SMRT sequencing adds to previously reported methods that employed the PacBio platform to interrogate clinically relevant low-complexity homologous regions of the human genome [26,27].

The *SLC6A4* gene encodes 5-HTT, which plays a key role in the central nervous system by regulating serotonergic signaling via transport of 5-HT from the synaptic cleft back into the pre-synaptic terminal for re-utilization. As such, *SLC6A4* has previously been associated with a range of behavioral and psychiatric disorders including depression, OCD, anxiety and schizophrenia [4]. Consistent with the role of 5-HTT in psychiatric phenotypes, variant *SLC6A4* alleles have also been implicated in interindividual SSRI response [5,6,7,8]. The principal *SLC6A4* variant associated with antidepressant efficacy is the 5-HTTLPR insertion/deletion polymorphism (rs4795541), which is located in the *SLC6A4* promoter and regulates gene expression. However, this region is challenging to interrogate given that the promoter is a GC rich variable number tandem repeat (VNTR) comprised of 11 to 24 homologous units.

The commonly studied 5-HTTLPR long (L) and short (S) alleles have variable frequencies across ancestral populations. Specifically, the 5-HTTLPR short (S) allele has a higher frequency in Asian populations (55–82%) [28], and a frequency in Europeans and Africans of 35–50% [28] and 22–25% [29,30], respectively. Although our study was not intended to measure population frequencies of the 5-HTTLPR L>S insertion/deletion polymorphism, long-read SMRT sequencing of 120 reference material samples detected a minor allele (S) frequency of 46.7%, which is consistent with a general population frequency.

In addition to 5-HTTLPR, the neighboring rs25531 (c.-1936A>G) variant has also been associated with reduced *SLC6A4* expression. Importantly, individuals who carry the 5-HTTLPR long (L) allele also have reduced *SLC6A4* expression when they concurrently harbor rs25531 c.-1936G [20,21], underscoring the importance of phasing *SLC6A4* variants into promoter haplotypes. Of note, the overall MAF of rs25531 in the gnomAD database is 0.1706 [31]; however, it is annotated as a low complexity region with a low genotype quality metric, and is only covered in fewer than 50% of individuals in gnomAD v2.1.1.

In addition to 5-HTTLPR and rs25531, rs25532 (c.-1760C>T) has also emerged as an independent determinant of *SLC6A4* expression. Notably, the rs25532 c.-1760T allele has been correlated with a 15–80% reduction in *SLC6A4* gene expression depending on the presence of other variants within the promoter haplotype [21]. Although the independent influence of rs25532 on *SLC6A4* expression is significant, it is most informative when taken into consideration as a haplotype with 5-HTTLPR and rs25531 [32]. The overall MAF of rs25532 in the gnomAD database is 0.0683 [31]; however, it is also annotated as a low complexity region. Interestingly, our analysis of 120 samples by long-read SMRT sequencing did not identify any individuals with the rs25532 c.-1760T allele *in cis* with the rs25531 c.-1936G allele, which is consistent with previous studies that suggested rs25532 c.-1760T is in linkage disequilibrium with rs25531 c.-1936A [21].

Short-read sequencing is not effective at accurately interrogating the *SLC6A4* promoter, particularly across the VNTR that includes the 5-HTTLPR insertion/deletion (L>S) polymorphism. This overarching limitation of short-read sequencing has previously been acknowledged, as low complexity regions and tandem repeats in the human genome are notoriously challenging for short-read platforms [33]. However, the ongoing improvements in long-read sequencing chemistry and throughput are increasingly enabling more accurate interrogation of these difficult regions for both research and clinical applications [34]. Our *SLC6A4* long-read SMRT sequencing results further support the integration of third-generation sequencing platforms for more accurate and comprehensive characterization of low-complexity pharmacogenetic regions.

In addition to interrogating homologous regions, another advantage of long-read SMRT sequencing is the ability to phase variants and define diplotypes over kilobases of genomic sequence. We previously leveraged long-read SMRT sequencing for full-length characterization of the *CYP2D6* gene [26], which led to the unambiguous detection of phased diplotypes and inferred phenotypes. Variant phasing is critical for more informed classification and interpretation in medical genetics, and defining full-gene haplotypes is an integral component of pharmacogenetics. The star (*) allele nomenclature system was developed over 20 years ago as a mechanism to characterize and report pharmacogenetic haplotypes, which is now centralized and administered by the NIH-funded Pharmacogene Variation (PharmVar) Consortium (https://www.pharmvar.org/) [35]. Although star (*) alleles have not yet been defined for *SLC6A4*, our long-read SMRT sequencing assay efficiently phased clinically relevant variants across the polymorphic homologous *SLC6A4* promoter, including the rare extra-long (XL) allele.

## 5. Conclusions

In conclusion, *SLC6A4* long-read SMRT sequencing is a reliable and validated third-generation sequencing technique that can accurately interrogate the low-complexity homologous *SLC6A4* promoter region. This novel method adds to previously reported long-read SMRT sequencing applications in pharmacogenomics, underscoring its utility for high-throughput variant detection and haplotype phasing. The capability to detect and phase the 5-HTTLPR L>S (rs4795541), rs25531 (c.-1936A>G), and rs25532 (c.-1760C>T) variants in the polymorphic *SLC6A4* promoter indicates that this method likely will have utility for both research and clinical testing applications.

## Figures and Tables

**Table 1:** Primer sequences used to amplify the *SLC6A4* promoter region.

Primer ID	Primer Sequence *	Amplicon Size (bp)
Forward primer		
SLC6A4F	5’-ATGGGTTCCAGAGTCAATCCTTTGCGTTTTCTGTTGCCCT-3’	-
Reverse primers		
SLC6A4R1	5’-GAAAGGTCTGGAGTCTTGATGAGGGACTGAGCTGGACAACCAC-3’	699
SLC6A4R2	5’-GAAAGGTCTGGAGTCTTGATCCCAGCAGGAGCCTATTGTT-3’	1026
SLC6A4R3	5’-GAAAGGTCTGGAGTCTTGATTCTCTTGACCTCGGACACCT-3’	1536
SLC6A4R4	5’-GAAAGGTCTGGAGTCTTGATGAAAGAAACGTGGGTTCGAGG-3’	2096

* Universal oligonucleotide tag sequence nucleotides are underlined.

**Table 2:** *SLC6A4* promoter haplotypes detected by SMRT sequencing.

	LAC	SAC	LGC	SAT	XLAC	SGC
*n* (freq) *	101 (0.421)	89 (0.371)	26 (0.108)	21 (0.088)	2 (0.008)	1 (0.004)

* Total *n* = 240 alleles. The haplotypes are defined by the rs4795541 (L/S)/rs25531 (A/G)/rs25532 (C/T) variants.

## Supplementary Materials

The following are available online at https://www.mdpi.com/2073-4425/11/11/1333/s1. Table S1: Summary of SLC6A4 Samples and Datasets. Table S2: Summary of SLC6A4 Promoter Sequencing Results. Table S3: Summary of Additional SLC6A4 Promoter Variants Detected by Long-Read SMRT Sequencing. Table S4: SLC6A4 Promoter Sanger Sequencing Confirmation. Table S5: Summary of SLC6A4 Long-Read SMRT Sequencing Precision. Figure S1: Representative Sanger sequencing results from selected SLC6A4promoter reference material samples.
