# Supplementary material for: Phased Haplotype Resolution of the SLC6A4 Promoter Using Long-Read Single Molecule Real-Time (SMRT) Sequencing
Source: Genes (Basel). 2020 Nov 12;11(11):1333. doi: 10.3390/genes11111333 (PMC7696006; doi:10.3390/genes11111333)

TABLE S1: Summary of SLC6A4 Samples and Datasets

n	Sample	WGS-1KG	WGS-GeT-RM	Capture Short Read	PacBio	PacBio 3-1-1	PacBio Diplotype	Sanger
1	HG00118	1	0	0	1	0	LAC/LAC	0
2	HG00130	1	0	0	1	0	LAC/SAC	0
3	HG00185	1	0	0	1	0	SAC/SAT	0
4	HG00246	1	0	0	1	0	LAC/SAC	0
5	HG00276	1	1	0	1	1	LAC/LGC	1
6	HG00332	1	0	0	1	0	LAC/SAC	0
7	HG00437	1	0	0	1	0	SAC/SAC	0
8	HG01083	1	0	0	1	0	LAC/SAC	0
9	HG01086	1	0	0	1	0	LAC/SAT	0
10	HG01089	1	0	0	1	1	SAC/SGC	0
11	HG01170	1	0	0	1	0	LAC/SAC	0
12	HG01359	1	0	0	1	0	LAC/SAT	0
13	HG01438	1	0	0	1	0	LAC/SAC	0
14	HG01566	1	0	0	1	0	LAC/SAC	0
15	HG01765	1	0	0	1	0	SAC/SAC	0
16	HG01845	1	0	0	1	0	SAC/SAC	0
17	HG02087	1	0	0	1	0	SAC/SAC	0
18	HG02649	1	0	0	1	0	SAC/SAC	0
19	HG03166	1	0	0	1	0	LAC/LGC	0
20	HG03225	1	0	0	1	0	LAC/SAC	0
21	HG03589	1	0	0	1	0	LAC/LAC	0
22	HG03624	1	0	0	1	0	LAC/SAC	0
23	NA12762	1	0	0	1	0	LAC/LAC	0
24	NA19315	1	0	0	1	0	LAC/LGC	0
25	NA19351	1	0	0	1	0	LGC/LGC	0
26	NA19395	1	0	0	1	0	LAC/LAC	0
27	NA19452	1	0	0	1	0	LAC/LAC	0
28	NA19466	1	0	0	1	0	LAC/LAC	0
29	NA20289	1	0	0	1	0	LGC/SAC	0
30	NA20356	1	0	0	1	0	SAC/SAC	0
31	NA20819	1	0	0	1	0	LAC/LAC	0
32	NA20901	1	0	0	1	0	LAC/SAC	0
33	NA12244	0	0	0	1	0	SAC/SAT	0
34	NA16688	0	0	0	1	0	LAC/SAC	0
35	NA17084	0	0	0	1	0	SAC/SAC	0
36	NA17222	0	0	0	1	0	LAC/SAC	0
37	NA17244	0	0	0	1	0	LAC/SAC	0
38	NA17246	0	0	0	1	0	LAC/LAC	0
39	NA17247	0	0	0	1	0	LAC/LGC	0
40	NA17280	0	0	0	1	0	SAT/SAT	0
41	NA17289	0	0	0	1	0	LAC/SAT	0
42	NA17296	0	0	0	1	0	LAC/SAC	0
43	NA07345	0	0	1	1	0	LAC/SAC	0
44	NA12750	0	0	1	1	0	LAC/SAT	0
45	NA12751	0	0	1	1	0	LAC/LAC	0
46	NA12802	0	0	1	1	0	LAC/LGC	0
47	NA12891	0	0	1	1	0	SAC/SAT	0
48	NA10860	0	0	1	1	0	LAC/SAC	0
49	NA10861	0	0	1	1	0	LGC/SAC	0
50	NA12155	0	0	1	1	0	SAC/SAT	0
51	NA12892	0	0	1	1	0	SAC/SAT	0
52	NA12336	0	0	1	1	0	LAC/LGC	1
53	NA12740	0	0	1	1	0	LAC/SAT	0
54	HG00436	0	1	0	1	1	LAC/LAC	0
55	HG00589	0	1	0	1	0	LAC/SAC	0
56	HG01190	0	1	1	1	1	LGC/SAC	1
57	NA06991	0	1	0	1	0	LAC/SAC	0
58	NA07000	0	1	0	1	1	LAC/SAT	1
59	NA07019	0	1	0	1	0	LAC/LAC	0
60	NA07029	0	1	0	1	1	SAT/SAT	0
61	NA07055	0	1	0	1	0	LAC/SAC	0
62	NA07056	0	1	0	1	0	LAC/LAC	0
63	NA07348	0	1	1	1	0	LAC/LAC	0
64	NA07357	0	1	1	1	0	LAC/LAC	0
65	NA10831	0	1	1	1	1	SAC/SAC	0
66	NA10847	0	1	0	1	1	SAC/SAT	0

67	NA10851	0	1	0	1	0	LAC/SAC	0
68	NA10854	0	1	1	1	0	SAT/SAT	0
69	NA11839	0	1	0	1	1	LAC/SAT	0
70	NA11993	0	1	0	1	0	LAC/LAC	0
71	NA12003	0	1	1	1	0	LAC/LAC	0
72	NA12006	0	1	0	1	0	LAC/SAC	0
73	NA12145	0	1	0	1	1	LAC/SAC	0
74	NA12156	0	1	1	1	0	LAC/SAC	0
75	NA12717	0	1	0	1	0	SAC/SAC	0
76	NA12813	0	1	0	1	0	SAC/SAT	0
77	NA12873	0	1	0	1	0	SAC/SAT	0
78	NA18484	0	1	0	1	0	LAC/SAC	0
79	NA18509	0	1	0	1	0	LAC/LGC	0
80	NA18518	0	1	0	1	0	LAC/LAC	0
81	NA18519	0	1	0	1	0	LAC/LGC	0
82	NA18524	0	1	0	1	0	LAC/SAC	0
83	NA18526	0	1	0	1	1	LGC/SAC	0
84	NA18540	0	1	0	1	0	SAC/SAC	0
85	NA18544	0	1	0	1	0	LGC/SAC	0
86	NA18552	0	1	0	1	0	SAC/SAC	0
87	NA18564	0	1	0	1	0	SAC/SAC	0
88	NA18565	0	1	0	1	0	LAC/LAC	0
89	NA18617	0	1	0	1	0	SAC/SAC	0
90	NA18855	0	1	0	1	1	LAC/XLAC	1
91	NA18861	0	1	0	1	0	LAC/LGC	0
92	NA18868	0	1	0	1	0	LAC/SAC	0
93	NA18942	0	1	0	1	1	LGC/SAC	0
94	NA18952	0	1	0	1	0	SAC/SAC	0
95	NA18959	0	1	0	1	0	SAC/SAC	0
96	NA18966	0	1	0	1	0	SAC/SAC	0
97	NA18973	0	1	0	1	0	SAC/SAC	0
98	NA18980	0	1	0	1	0	SAC/SAC	0
99	NA18992	0	1	0	1	0	LAC/LGC	0
100	NA19003	0	1	0	1	0	SAC/SAC	0
101	NA19007	0	1	0	1	0	LAC/SAC	0
102	NA19095	0	1	0	1	0	LAC/LAC	0
103	NA19109	0	1	0	1	0	LAC/LGC	0
104	NA19122	0	1	0	1	0	LAC/LAC	0
105	NA19143	0	1	0	1	0	LAC/LGC	0
106	NA19147	0	1	0	1	0	LGC/SAC	0
107	NA19174	0	1	0	1	1	SAC/XLAC	1
108	NA19176	0	1	0	1	1	LGC/LGC	0
109	NA19178	0	1	0	1	0	LAC/LGC	0
110	NA19207	0	1	0	1	0	LAC/SAC	0
111	NA19226	0	1	0	1	0	LAC/SAC	0
112	NA19239	0	1	1	1	0	LAC/LAC	0
113	NA19789	0	1	1	1	0	LAC/LGC	0
114	NA19819	0	1	1	1	0	LAC/SAC	0
115	NA19908	0	1	0	1	0	SAC/SAC	0
116	NA19917	0	1	0	1	0	LAC/LAC	0
117	NA19920	0	1	0	1	0	LAC/SAC	0
118	NA20296	0	1	0	1	0	LAC/LGC	0
119	NA20509	0	1	0	1	0	SAC/SAC	0
120	NA21781	0	1	0	1	0	LAC/LAC	0

TABLE S2: Summary of SLC6A4 Promoter Sequencing Results

		1KG Genotype			GeT-RM Genotype			Short-Read Capture Genotype			PacBio Genotype			
n	Sample	rs4795541	rs25531	rs25532	rs4795541	rs25531	rs25532	rs4795541	rs25531	rs25532	rs4795541	rs25531	rs25532	PacBio Diplotype
1	HG00118	./.	./.	CC	-	-	-	-	-	-	LL	AA	CC	LAC/LAC
2	HG00130	./.	./.	./.	-	-	-	-	-	-	LS	AA	CC	LAC/SAC
3	HG00185	./.	AA	./.	-	-	-	-	-	-	SS	AA	CT	SAC/SAT
4	HG00246	./.	./.	./.	-	-	-	-	-	-	LS	AA	CC	LAC/SAC
5	HG00276	./.	./.	./.	./.	AG	CC	-	-	-	LL	AG	CC	LAC/LGC
6	HG00332	./.	./.	CC	-	-	-	-	-	-	LS	AA	CC	LAC/SAC
7	HG00437	./.	./.	./.	-	-	-	-	-	-	SS	AA	CC	SAC/SAC
8	HG01083	./.	./.	./.	-	-	-	-	-	-	LS	AA	CC	LAC/SAC
9	HG01086	./.	./.	./.	-	-	-	-	-	-	LS	AA	CT	LAC/SAT
10	HG01089	./.	AA	CC	-	-	-	-	-	-	SS	AG	CC	SAC/SGC
11	HG01170	./.	./.	./.	-	-	-	-	-	-	LS	AA	CC	LAC/SAC
12	HG01359	./.	./.	./.	-	-	-	-	-	-	LS	AA	CT	LAC/SAT
13	HG01438	./.	./.	CC	-	-	-	-	-	-	LS	AA	CC	LAC/SAC
14	HG01566	./.	AA	./.	-	-	-	-	-	-	LS	AA	CC	LAC/SAC
15	HG01765	./.	./.	./.	-	-	-	-	-	-	SS	AA	CC	SAC/SAC
16	HG01845	./.	./.	./.	-	-	-	-	-	-	SS	AA	CC	SAC/SAC
17	HG02087	LL	./.	./.	-	-	-	-	-	-	SS	AA	CC	SAC/SAC
18	HG02649	./.	./.	./.	-	-	-	-	-	-	SS	AA	CC	SAC/SAC
19	HG03166	./.	./.	./.	-	-	-	-	-	-	LL	AG	CC	LAC/LGC
20	HG03225	./.	./.	./.	-	-	-	-	-	-	LS	AA	CC	LAC/SAC
21	HG03589	./.	./.	./.	-	-	-	-	-	-	LL	AA	CC	LAC/LAC
22	HG03624	./.	./.	./.	-	-	-	-	-	-	LS	AA	CC	LAC/SAC
23	NA12762	./.	./.	./.	-	-	-	-	-	-	LL	AA	CC	LAC/LAC
24	NA19315	./.	./.	./.	-	-	-	-	-	-	LL	AG	CC	LAC/LGC
25	NA19351	./.	./.	./.	-	-	-	-	-	-	LL	GG	CC	LGC/LGC
26	NA19395	./.	./.	./.	-	-	-	-	-	-	LL	AA	CC	LAC/LAC
27	NA19452	./.	./.	./.	-	-	-	-	-	-	LL	AA	CC	LAC/LAC
28	NA19466	./.	./.	./.	-	-	-	-	-	-	LL	AA	CC	LAC/LAC
29	NA20289	./.	AA	./.	-	-	-	-	-	-	LS	AG	CC	LGC/SAC
30	NA20356	./.	./.	./.	-	-	-	-	-	-	SS	AA	CC	SAC/SAC
31	NA20819	./.	./.	CC	-	-	-	-	-	-	LL	AA	CC	LAC/LAC
32	NA20901	./.	./.	CC	-	-	-	-	-	-	LS	AA	CC	LAC/SAC
33	NA12244	-	-	-	-	-	-	-	-	-	SS	AA	CT	SAC/SAT
34	NA16688	-	-	-	-	-	-	-	-	-	LS	AA	CC	LAC/SAC
35	NA17084	-	-	-	-	-	-	-	-	-	SS	AA	CC	SAC/SAC
36	NA17222	-	-	-	-	-	-	-	-	-	LS	AA	CC	LAC/SAC
37	NA17244	-	-	-	-	-	-	-	-	-	LS	AA	CC	LAC/SAC
38	NA17246	-	-	-	-	-	-	-	-	-	LL	AA	CC	LAC/LAC
39	NA17247	-	-	-	-	-	-	-	-	-	LL	AG	CC	LAC/LGC
40	NA17280	-	-	-	-	-	-	-	-	-	SS	AA	TT	SAT/SAT
41	NA17289	-	-	-	-	-	-	-	-	-	LS	AA	CT	LAC/SAT
42	NA17296	-	-	-	-	-	-	-	-	-	LS	AA	CC	LAC/SAC
43	NA07345	-	-	-	-	-	-	./.	./.	./.	LS	AA	CC	LAC/SAC
44	NA12750	-	-	-	-	-	-	LS	AA	CT	LS	AA	CT	LAC/SAT
45	NA12751	-	-	-	-	-	-	LL	AA	./.	LL	AA	CC	LAC/LAC
46	NA12802	-	-	-	-	-	-	./.	AG	./.	LL	AG	CC	LAC/LGC
47	NA12891	-	-	-	-	-	-	SS	./.	CT	SS	AA	CT	SAC/SAT
48	NA10860	-	-	-	-	-	-	./.	AA	./.	LS	AA	CC	LAC/SAC
49	NA10861	-	-	-	-	-	-	./.	AG	./.	LS	AG	CC	LGC/SAC
50	NA12155	-	-	-	-	-	-	LL	AA	CT	SS	AA	CT	SAC/SAT
51	NA12892	-	-	-	-	-	-	SS	AA	CT	SS	AA	CT	SAC/SAT
52	NA12336	-	-	-	-	-	-	./.	AG	./.	LL	AG	CC	LAC/LGC
53	NA12740	-	-	-	-	-	-	LS	AA	CT	LS	AA	CT	LAC/SAT
54	HG00436	-	-	-	./.	./.	CC	-	-	-	LL	AA	CC	LAC/LAC
55	HG00589	-	-	-	SS	./.	./.	-	-	-	LS	AA	CC	LAC/SAC
56	HG01190	-	-	-	./.	GG	./.	./.	AG	./.	LS	AG	CC	LGC/SAC
57	NA06991	-	-	-	LS	./.	./.	-	-	-	LS	AA	CC	LAC/SAC
58	NA07000	-	-	-	./.	./.	CT	-	-	-	LS	AA	CT	LAC/SAT
59	NA07019	-	-	-	./.	AA	CC	-	-	-	LL	AA	CC	LAC/LAC
60	NA07029	-	-	-	SS	./.	TT	-	-	-	SS	AA	TT	SAT/SAT
61	NA07055	-	-	-	LS	./.	CC	-	-	-	LS	AA	CC	LAC/SAC
62	NA07056	-	-	-	./.	./.	CC	-	-	-	LL	AA	CC	LAC/LAC
63	NA07348	-	-	-	LL	./.	CC	./.	AA	./.	LL	AA	CC	LAC/LAC
64	NA07357	-	-	-	./.	./.	./.	./.	AA	./.	LL	AA	CC	LAC/LAC
65	NA10831	-	-	-	SS	./.	CC	SS	./.	./.	SS	AA	CC	SAC/SAC
66	NA10847	-	-	-	SS	AA	CT	-	-	-	SS	AA	CT	SAC/SAT
67	NA10851	-	-	-	./.	./.	./.	-	-	-	LS	AA	CC	LAC/SAC
68	NA10854	-	-	-	SS	./.	TT	./.	AA	TT	SS	AA	TT	SAT/SAT
69	NA11839	-	-	-	./.	./.	./.	-	-	-	LS	AA	CT	LAC/SAT
70	NA11993	-	-	-	LL	./.	CC	-	-	-	LL	AA	CC	LAC/LAC
71	NA12003	-	-	-	./.	./.	CC	./.	AA	./.	LL	AA	CC	LAC/LAC
72	NA12006	-	-	-	LS	./.	CC	-	-	-	LS	AA	CC	LAC/SAC
73	NA12145	-	-	-	LS	AA	CC	-	-	-	LS	AA	CC	LAC/SAC
74	NA12156	-	-	-	./.	./.	CC	LL	./.	./.	LS	AA	CC	LAC/SAC
75	NA12717	-	-	-	SS	./.	CC	-	-	-	SS	AA	CC	SAC/SAC
76	NA12813	-	-	-	SS	./.	CT	-	-	-	SS	AA	CT	SAC/SAT
77	NA12873	-	-	-	LS	./.	CT	-	-	-	SS	AA	CT	SAC/SAT
78	NA18484	-	-	-	LS	AA	./.	-	-	-	LS	AA	CC	LAC/SAC
79	NA18509	-	-	-	./.	AG	CC	-	-	-	LL	AG	CC	LAC/LGC
80	NA18518	-	-	-	LL	./.	CC	-	-	-	LL	AA	CC	LAC/LAC
81	NA18519	-	-	-	./.	AG	./.	-	-	-	LL	AG	CC	LAC/LGC
82	NA18524	-	-	-	LS	./.	CC	-	-	-	LS	AA	CC	LAC/SAC
83	NA18526	-	-	-	./.	AG	CC	-	-	-	LS	AG	CC	LGC/SAC
84	NA18540	-	-	-	SS	./.	CC	-	-	-	SS	AA	CC	SAC/SAC
85	NA18544	-	-	-	LS	./.	CC	-	-	-	LS	AG	CC	LGC/SAC
86	NA18552	-	-	-	SS	AA	CC	-	-	-	SS	AA	CC	SAC/SAC
87	NA18564	-	-	-	SS	./.	CC	-	-	-	SS	AA	CC	SAC/SAC
88	NA18565	-	-	-	./.	./.	CC	-	-	-	LL	AA	CC	LAC/LAC
89	NA18617	-	-	-	SS	./.	CC	-	-	-	SS	AA	CC	SAC/SAC
90	NA18855	-	-	-	LL	./.	CC	-	-	-	LXL	AA	CC	LAC/XLAC
91	NA18861	-	-	-	./.	AG	CC	-	-	-	LL	AG	CC	LAC/LGC
92	NA18868	-	-	-	LS	AA	CC	-	-	-	LS	AA	CC	LAC/SAC
93	NA18942	-	-	-	LL	GG	CC	-	-	-	LS	AG	CC	LGC/SAC
94	NA18952	-	-	-	SS	./.	CC	-	-	-	SS	AA	CC	SAC/SAC
95	NA18959	-	-	-	SS	./.	CC	-	-	-	SS	AA	CC	SAC/SAC
96	NA18966	-	-	-	SS	./.	CC	-	-	-	SS	AA	CC	SAC/SAC
97	NA18973	-	-	-	SS	./.	CC	-	-	-	SS	AA	CC	SAC/SAC
98	NA18980	-	-	-	SS	AA	CC	-	-	-	SS	AA	CC	SAC/SAC
99	NA18992	-	-	-	./.	AG	./.	-	-	-	LL	AG	CC	LAC/LGC
100	NA19003	-	-	-	SS	./.	./.	-	-	-	SS	AA	CC	SAC/SAC
101	NA19007	-	-	-	LS	./.	CC	-	-	-	LS	AA	CC	LAC/SAC
102	NA19095	-	-	-	./.	./.	CC	-	-	-	LL	AA	CC	LAC/LAC
103	NA19109	-	-	-	./.	AG	CC	-	-	-	LL	AG	CC	LAC/LGC

104	NA19122	-	-	-	LL	./.	CC	-	-	-	LL	AA	CC	LAC/LAC
105	NA19143	-	-	-	LL	AG	CC	-	-	-	LL	AG	CC	LAC/LGC
106	NA19147	-	-	-	LS	./.	CC	-	-	-	LS	AG	CC	LGC/SAC
107	NA19174	-	-	-	LS	./.	CC	-	-	-	SXL	AA	CC	SAC/XLAC
108	NA19176	-	-	-	LL	GG	./.	-	-	-	LL	GG	CC	LGC/LGC
109	NA19178	-	-	-	LL	AG	CC	-	-	-	LL	AG	CC	LAC/LGC
110	NA19207	-	-	-	LS	./.	CC	-	-	-	LS	AA	CC	LAC/SAC
111	NA19226	-	-	-	LS	./.	CC	-	-	-	LS	AA	CC	LAC/SAC
112	NA19239	-	-	-	./.	./.	CC	LL	AA	CC	LL	AA	CC	LAC/LAC
113	NA19789	-	-	-	LL	AG	CC	./.	./.	./.	LL	AG	CC	LAC/LGC
114	NA19819	-	-	-	LS	./.	CC	./.	./.	./.	LS	AA	CC	LAC/SAC
115	NA19908	-	-	-	SS	./.	CC	-	-	-	SS	AA	CC	SAC/SAC
116	NA19917	-	-	-	LL	./.	CC	-	-	-	LL	AA	CC	LAC/LAC
117	NA19920	-	-	-	LS	./.	CC	-	-	-	LS	AA	CC	LAC/SAC
118	NA20296	-	-	-	./.	AG	CC	-	-	-	LL	AG	CC	LAC/LGC
119	NA20509	-	-	-	SS	./.	CC	-	-	-	SS	AA	CC	SAC/SAC
120	NA21781	-	-	-	LL	./.	CC	-	-	-	LL	AA	CC	LAC/LAC

Red characters: Discordant nucleotide from long-read SMRT sequencing.

TABLE S3: Summary of Additional SLC6A4 Promoter Variants Detected by Long-Read SMRT Sequencing

n	Sample	rs76568780	rs77638792	rs56087640	rs1408618839	rs25530	rs1462091560
1	HG00276	-	-	-	CT	-	-
2	NA19395	-	-	AT	-	-	-
3	HG00436	AA	AA	-	-	-	A/delA
4	NA12145	-	-	-	-	CT	-
5	NA18484	-	-	AT	-	-	-
6	NA18509	-	-	AT	-	-	-
7	NA18524	GA	GA	-	-	-	-
8	NA18565	AA	AA	-	-	-	-
9	NA18992	GA	GA	-	-	-	-
10	NA19122	-	-	AT	-	-	-
11	NA19178	GA	GA	-	-	-	-
12	NA19917	GA	GA	-	-	-	-

TABLE S4: Summary of SLC6A4 Promoter Sanger Sequencing Confirmation

		PacBio Genotype				Sanger Genotype		
n	Sample	rs4795541	rs25531	rs25532	PacBio Diplotype	rs47955412	rs255313	rs255324
1	HG00276	LL	AG	CC	LAC/LGC	LL	AG	CC
2	NA12336	LL	AG	CC	LAC/LGC	LL	AG	CC
3	HG01190	LS	AG	CC	LGC/SAC	LS	AG	CC
4	NA07000	LS	AA	CT	LAC/SAT	LS	AA	CT
5	NA18855	LXL	AA	CC	LAC/XLAC	LXL	AA	CC
6	NA19174	SXL	AA	CC	SAC/XLAC	SXL	AA	CC

TABLE S5: Summary of SLC6A4 Long-Read SMRT Sequencing Reproducibility

						Additional SLC6A4 Promoter Variants Detected by Long-Read SMRT Sequencing					
n	Sample	rs4795541	rs25531	rs25532	Diplotype	rs76568780	rs77638792	rs56087640	rs1408618839	rs25530	rs1462091560
1	HG00276_1a	LL	AG	CC	LAC/LGC	-	-	-	CT	-	-
2	HG00276_1b	LL	AG	CC	LAC/LGC	-	-	-	CT	-	-
3	HG00276_1c	LL	AG	CC	LAC/LGC	-	-	-	CT	-	-
4	HG00276_2	LL	AG	CC	LAC/LGC	-	-	-	CT	-	-
5	HG00276_3	LL	AG	CC	LAC/LGC	-	-	-	CT	-	-
6	HG00436_1a	LL	AA	CC	LAC/LAC	AA	AA	-	-	-	A/delA
7	HG00436_1b	LL	AA	CC	LAC/LAC	AA	AA	-	-	-	A/delA
8	HG00436_1c	LL	AA	CC	LAC/LAC	AA	AA	-	-	-	A/delA
9	HG00436_2	LL	AA	CC	LAC/LAC	AA	AA	-	-	-	A/delA
10	HG00436_3	LL	AA	CC	LAC/LAC	AA	AA	-	-	-	A/delA
11	HG01190_1a	LS	AG	CC	LGC/SAC	-	-	-	-	-	-
12	HG01190_1b	LS	AG	CC	LGC/SAC	-	-	-	-	-	-
13	HG01190_1c	LS	AG	CC	LGC/SAC	-	-	-	-	-	-
14	HG01190_2	LS	AG	CC	LGC/SAC	-	-	-	-	-	-
15	HG01190_3	LS	AG	CC	LGC/SAC	-	-	-	-	-	-
16	NA07000_1a	LS	AA	CT	LAC/SAT	-	-	-	-	-	-
17	NA07000_1b	LS	AA	CT	LAC/SAT	-	-	-	-	-	-
18	NA07000_1c	LS	AA	CT	LAC/SAT	-	-	-	-	-	-
19	NA07000_2	LS	AA	CT	LAC/SAT	-	-	-	-	-	-
20	NA07000_3	LS	AA	CT	LAC/SAT	-	-	-	-	-	-
21	NA07029_1a	SS	AA	TT	SAT/SAT	-	-	-	-	-	-
22	NA07029_1b	SS	AA	TT	SAT/SAT	-	-	-	-	-	-
23	NA07029_1c	SS	AA	TT	SAT/SAT	-	-	-	-	-	-
24	NA07029_2	SS	AA	TT	SAT/SAT	-	-	-	-	-	-
25	NA07029_3	SS	AA	TT	SAT/SAT	-	-	-	-	-	-
26	NA10831_1a	SS	AA	CC	SAC/SAC	-	-	-	-	-	-
27	NA10831_1b	SS	AA	CC	SAC/SAC	-	-	-	-	-	-
28	NA10831_1c	SS	AA	CC	SAC/SAC	-	-	-	-	-	-
29	NA10831_2	SS	AA	CC	SAC/SAC	-	-	-	-	-	-
30	NA10831_3	SS	AA	CC	SAC/SAC	-	-	-	-	-	-
31	NA10847_1a	SS	AA	CT	SAC/SAT	-	-	-	-	-	-
32	NA10847_1b	SS	AA	CT	SAC/SAT	-	-	-	-	-	-
33	NA10847_1c	SS	AA	CT	SAC/SAT	-	-	-	-	-	-
34	NA10847_2	SS	AA	CT	SAC/SAT	-	-	-	-	-	-
35	NA10847_3	SS	AA	CT	SAC/SAT	-	-	-	-	-	-
36	NA11839_1a	LS	AA	CT	LAC/SAT	-	-	-	-	-	-
37	NA11839_1b	LS	AA	CT	LAC/SAT	-	-	-	-	-	-
38	NA11839_1c	LS	AA	CT	LAC/SAT	-	-	-	-	-	-
39	NA11839_2	LS	AA	CT	LAC/SAT	-	-	-	-	-	-
40	NA11839_3	LS	AA	CT	LAC/SAT	-	-	-	-	-	-
41	NA12145_1a	LS	AA	CC	LAC/SAC	-	-	-	-	CT	-
42	NA12145_1b	LS	AA	CC	LAC/SAC	-	-	-	-	CT	-
43	NA12145_1c	LS	AA	CC	LAC/SAC	-	-	-	-	CT	-
44	NA12145_2	LS	AA	CC	LAC/SAC	-	-	-	-	CT	-
45	NA12145_3	LS	AA	CC	LAC/SAC	-	-	-	-	CT	-
46	NA18526_1a	LS	AG	CC	LGC/SAC	-	-	-	-	-	-
47	NA18526_1b	LS	AG	CC	LGC/SAC	-	-	-	-	-	-
48	NA18526_1c	LS	AG	CC	LGC/SAC	-	-	-	-	-	-
49	NA18526_2	LS	AG	CC	LGC/SAC	-	-	-	-	-	-
50	NA18526_3	LS	AG	CC	LGC/SAC	-	-	-	-	-	-
51	NA18855_1a	LXL	AA	CC	LAC/XLAC	-	-	-	-	-	-
52	NA18855_1b	LXL	AA	CC	LAC/XLAC	-	-	-	-	-	-
53	NA18855_1c	LXL	AA	CC	LAC/XLAC	-	-	-	-	-	-
54	NA18855_2	LXL	AA	CC	LAC/XLAC	-	-	-	-	-	-
55	NA18855_3	LXL	AA	CC	LAC/XLAC	-	-	-	-	-	-
56	NA18942_1a	LS	AG	CC	LGC/SAC	-	-	-	-	-	-
57	NA18942_1b	LS	AG	CC	LGC/SAC	-	-	-	-	-	-
58	NA18942_1c	LS	AG	CC	LGC/SAC	-	-	-	-	-	-
59	NA18942_2	LS	AG	CC	LGC/SAC	-	-	-	-	-	-
60	NA18942_3	LS	AG	CC	LGC/SAC	-	-	-	-	-	-
61	NA19174_1a	SXL	AA	CC	SAC/XLAC	-	-	-	-	-	-
62	NA19174_1b	SXL	AA	CC	SAC/XLAC	-	-	-	-	-	-
63	NA19174_1c	SXL	AA	CC	SAC/XLAC	-	-	-	-	-	-
64	NA19174_2	SXL	AA	CC	SAC/XLAC	-	-	-	-	-	-
65	NA19174_3	SXL	AA	CC	SAC/XLAC	-	-	-	-	-	-
66	NA19176_1a	LL	GG	CC	LGC/LGC	-	-	-	-	-	-
67	NA19176_1b	LL	GG	CC	LGC/LGC	-	-	-	-	-	-
68	NA19176_1c	LL	GG	CC	LGC/LGC	-	-	-	-	-	-
69	NA19176_2	LL	GG	CC	LGC/LGC	-	-	-	-	-	-
70	NA19176_3	LL	GG	CC	LGC/LGC	-	-	-	-	-	-
71	HG01089_1a	SS	AG	CC	SAC/SGC	-	-	-	-	-	-
72	HG01089_1b	SS	AG	CC	SAC/SGC	-	-	-	-	-	-
73	HG01089_1c	SS	AG	CC	SAC/SGC	-	-	-	-	-	-
74	HG01089_2	SS	AG	CC	SAC/SGC	-	-	-	-	-	-
75	HG01089_3	SS	AG	CC	SAC/SGC	-	-	-	-	-	-

Supplemental Figure S1:
Representative Sanger sequencing results from selected *SLC6A4* promoter reference material samples.

HG00276: LAC/LGC

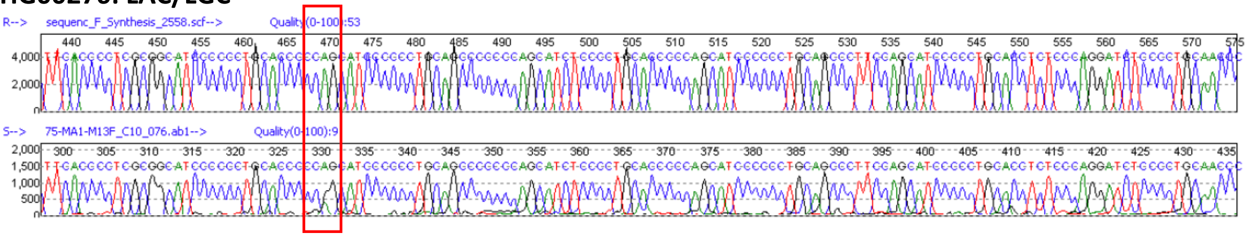

HG01190: LGC/SAC

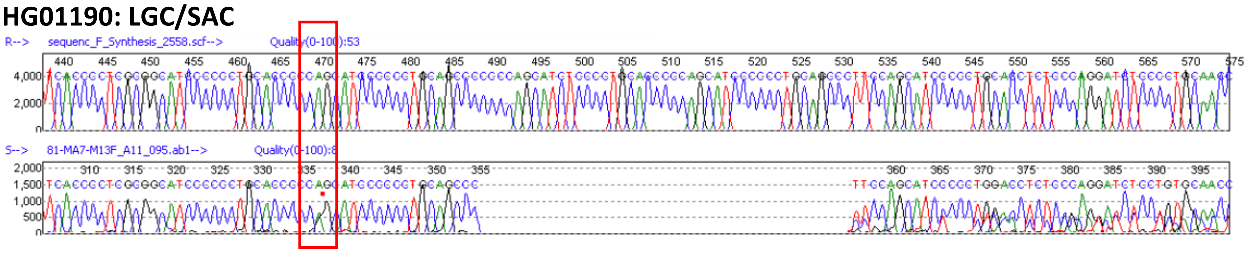

NA07000: LAC/SAT

Fwd:

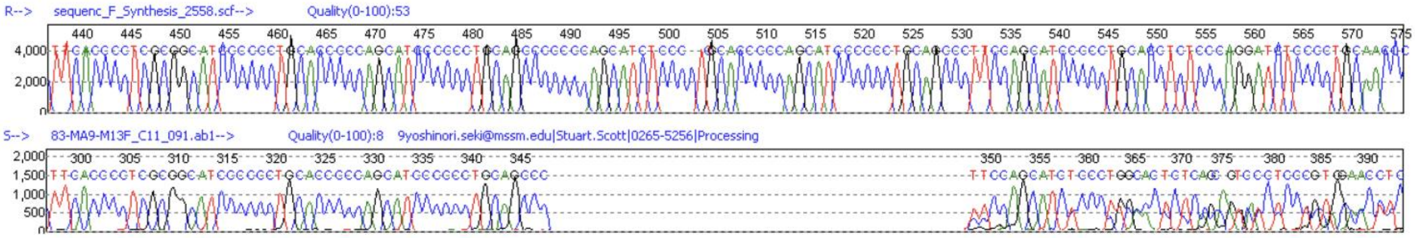

Rev:

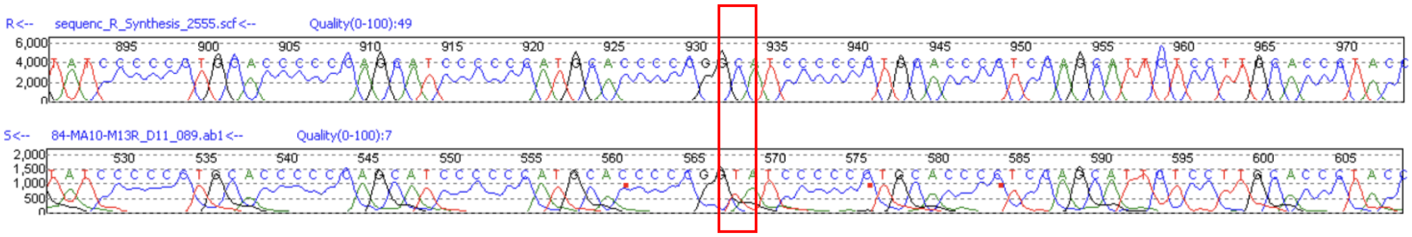

NA19174: SAC/XLAC

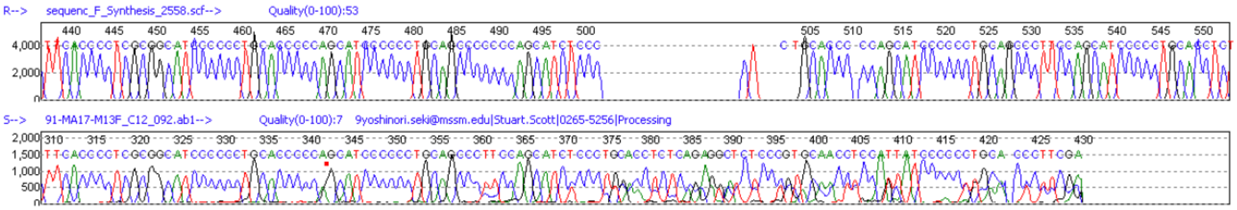

Supplement: Supplementary file 1 [file genes-11-01333-s001.pdf]
